# Impact of acute kidney injury in elderly versus young deceased donors on post-transplant outcomes: A multicenter cohort study

**DOI:** 10.1038/s41598-020-60726-8

**Published:** 2020-02-28

**Authors:** Woo Yeong Park, Jeong Ho Kim, Eun Jung Ko, Ji-Won Min, Tae Hyun Ban, Hye-Eun Yoon, Young Soo Kim, Kyubok Jin, Seungyeup Han, Chul Woo Yang, Byung Ha Chung

**Affiliations:** 1 Transplant research center, Seoul, Republic of Korea; 2 Division of Nephrology, Department of Internal Medicine, Seoul St. Mary’s Hospital, College of Medicine, The Catholic University of Korea, Seoul, Republic of Korea; 3 Division of Nephrology, Department of Internal Medicine, Daejeon St. Mary’s hospital, College of Medicine, The Catholic University of Korea, Daejeon, Republic of Korea; 4 Division of Nephrology, Department of Internal Medicine, Bucheon St. Mary’s hospital, College of Medicine, The Catholic University of Korea, Bucheon, Republic of Korea; 5 Division of Nephrology, Department of Internal Medicine, Eunpyeong St. Mary’s hospital, College of Medicine, The Catholic University of Korea, Seoul, Republic of Korea; 6 Division of Nephrology, Department of Internal Medicine, Incheon St. Mary’s hospital, College of Medicine, The Catholic University of Korea, Incheon, Republic of Korea; 7 Division of Nephrology, Department of Internal Medicine, Uijeongbu St. Mary’s hospital, College of Medicine, The Catholic University of Korea, Uijeongbu, Republic of Korea; 8 Department of Internal Medicine, Keimyung University School of Medicine, Daegu, Republic of Korea; 9 Keimyung University Kidney Institute, Daegu, Republic of Korea

**Keywords:** Acute kidney injury, End-stage renal disease

## Abstract

We investigated the impact of acute kidney injury (AKI) in elderly deceased-donors (DDs) vs. AKI in young DDs on post-transplant clinical outcomes. A total of 709 kidney transplant recipients (KTRs) from 602 DDs at four transplant centers were enrolled. KTRs were divided into young-DDKT and elderly-DDKT groups according to the age of DD of 60 years. Both groups were subdivided into non-AKI-KT and AKI-KT subgroups according to AKI in DDs. We investigated short-term and long-term clinical outcomes of non-AKI-DDKT and AKI-DDKT subgroups within young-DDKT and elderly-DDKT groups. The incidence of DGF in the AKI-DDKT subgroup was higher and the allograft function within 12 months after KT in the AKI-DDKT subgroup was lower than those in the non-AKI-DDKT subgroup in both young-DDKT and elderly-DDKT groups. Death-censored allograft survival rate was significantly lower in the AKI-elderly-DDKT subgroup than that in the non-AKI-elderly-DDKT subgroup, but it did not differ between AKI-young-DDKT and non-AKI-young-DDKT subgroup. In multivariable analysis, AKI-elderly-DDKT was an independent risk factor for allograft failure (hazard ratio: 2.648, 95% CI: 1.170–5.994, *p* = 0.019) and a significant interaction between AKI and old age in DDs on allograft failure was observed (*p* = 0.001). AKI in elderly DDs, but not in young DDs, can significantly affect long-term allograft outcomes of KTRs.

## Introduction

With a tremendous increase in the number of patients with end-stage renal disease, donor shortage in kidney transplantation (KT) has become a worldwide crucial issue to solve^1–3^. In this regard, the use of kidneys from elderly deceased donors (DDs) has been proposed as an important strategy for solving this donor shortage^4–7^. It has been reported that the proportion of elderly brain death patients was higher than that of younger brain death patients and that KT from elderly DDs could give survival benefit in comparison with those remaining on dialysis^8^. In addition, in contrast to living donor KT, kidney donation from elderly DD is fully free from donor safety issue. These reasons justify the use of kidney from elderly DDs.

Acute kidney injury (AKI) is very commonly detected in individuals with brain death state for various reasons^9,10^. Donor shortage also has driven the use of kidney from DDs with AKI. Previous studies including our own have shown that the long-term allograft outcome of KT from DDs with AKI is not significantly different from that of KT from DDs without AKI^11–14^. However, with respect to transplant-naive individuals, long-term renal outcomes after AKI episode depend on the underlying status of the kidney. Renal outcomes of AKI in elderly who are supposed to have underlying chronic kidney injury despite normal serum creatinine level are significantly inferior to those of AKI in younger individuals^15,16^. Therefore, AKI in elderly-DDs might have adverse impact on long-term post-transplant allograft outcomes in their corresponding recipients. However, this has not been fully investigated yet.

Based on these backgrounds, the aim of this study was to investigate whether the impact of AKI in elderly DDs on post-transplant clinical outcomes might differ from that of AKI in young-DDs. For this, short-term and long-term clinical outcomes of KT from DDs without AKI (non-AKI-DDKT subgroup) versus those for KT from DDs with AKI (AKI-DDKT subgroup) within young-DDKT and elderly-DDKT groups were compared. The interaction between AKI and old age in DDs on post-transplant allograft survival was also investigated.

## Results

### Comparison of baseline characteristics between young-DDKT and elderly-DDKT groups and between non-AKI and AKI subgroups within each group

The median follow-up period of this study was 62.5 (interquartile range: 42.3–91.7) months. The proportion of HTN was significantly higher in the elderly DD group than that in the young DD group (41.8% vs. 17.0%, *p* < 0.001). There was no significant difference in the proportion of AKI or the distribution of AKI severity between young DD and elderly DD groups. KDPI score was significantly higher in the elderly-DDs than that in the young-DDs. In corresponding recipients, the mean age and the proportion of DM as primary renal disease were higher in the elderly-DDKT group than those in the young-DDKT group (63.6 ± 3.0 vs. 46.7 ± 8.5 years, *p* < 0.001; 27.0% vs. 18.9%, *p* = 0.054). The proportion of re-transplants was significantly lower in the elderly-DDKT group than that in the young-DDKT group (5.4% vs. 11.9%, *p* = 0.046) (see Supplementary Table S1).

In the subgroup analysis, mean donor age, BMI, the proportion of cause of donor death by CVA, and KDPI score were significantly higher in the group of young DDs. Baseline eGFR was significantly lower in the subgroup of AKI DDs than that in the subgroup of non-AKI DDs. However, in the group of elderly DDs, there were no significant differences in donor age, gender, BMI, HTN, DM, cause of donor death by CVA, or baseline GFR between non-AKI DDs and AKI DDs subgroups. KDPI score was higher in AKI-DDs subgroup than that in the non-AKI-DDs subgroup in both young- and elderly-DDKT group. In corresponding recipients, proportions of anti-thymocyte globulin were significantly higher in young-DDKT and elderly-DDKT groups with AKI in comparison with those without AKI (33.8% vs. 18.2%, *p* < 0.001 and 43.8% vs. 19.1%, *p* = 0.008, respectively) (Table 1).

**Table 1:** Comparison of clinical and laboratory parameters according to acute kidney injury in young- or elderly donors.

Variables	Young-DDKT			Elderly-DDKT		
	Non-AKI-KT	AKI-KT	p for Trend	Non-AKI-KT	AKI-KT	p for Trend
**Donors**	n = 246	n = 276		n = 34	n = 46	
Age at KT (years)	39.2 ± 14.2	44.3 ± 11.8	<0.001	63.9 ± 3.7	63.8 ± 3.5	0.916
Gender (Male: Female)	151: 95	209: 67	<0.001	20: 14	33: 13	0.243
Body mass index (kg/m^2^)	22.4 ± 3.8	23.8 ± 3.6	<0.001	24.1 ± 3.1	22.9 ± 2.7	0.088
Hypertension, n (%)	42 (17.4)	46 (16.7)	0.816	14 (42.4)	19 (41.3)	1.000
Diabetes mellitus, n (%)	20 (8.3)	22 (8.0)	1.000	2 (6.1)	10 (21.7)	0.065
Cause of donor death - CVA, n (%)	154 (62.6)	212 (76.8)	<0.001	27 (79.4)	31 (67.4)	0.125
Baseline GFR (ml/min/1.73 m^2^) (CKD-EPI)	86.8 ± 30.2	82.4 ± 21.1	0.100	76.4 ± 21.9	75.7 ± 13.3	0.898
GFR at allocation (ml/min/1.73 m^2^) (CKD-EPI)	102.5 ± 31.5	40.0 ± 23.3	<0.001	81.2 ± 24.0	34.5 ± 20.7	<0.001
KDPI score (%)	50.3 ± 22.7	65.4 ± 19.8	<0.001	91.3 ± 5.4	94.4 ± 4.9	0.011
**Recipients**	n = 264	n = 334		n = 47	n = 64	
Transplant year, n (%)			0.060			0.808
1996 ~ 2005	18 (6.8)	18 (5.4)		3 (6.4)	2 (3.1)	
2006 ~ 2010	50 (18.9)	45 (13.5)		3 (6.4)	4 (6.3)	
2011 ~ 2017	196 (74.2)	271 (81.1)		41 (87.2)	58 (90.6)	
Age at KT (yr)	47.5 ± 9.7	48.7 ± 10.2	0.131	55.1 ± 9.5	54.2 ± 7.6	0.591
Gender (Male: Female)	156: 108	193: 141	0.802	30: 17	39: 25	0.844
Body mass index (kg/m^2^)	22.9 ± 3.4	23.2 ± 4.1	0.261	23.6 ± 3.6	23.6 ± 3.6	0.974
Hypertension, n (%)	215 (81.4)	285 (85.3)	0.222	41 (87.2)	55 (85.9)	1.000
Diabetes mellitus, n (%)	45 (17.0)	68 (20.4)	0.344	11 (23.4)	19 (29.7)	0.521
Dialysis duration, years	8.3 ± 8.3	8.2 ± 10.8	0.924	6.4 ± 5.2	8.4 ± 14.5	0.354
Previous KT, n (%)	27 (10.2)	44 (13.2)	0.309	1 (2.1)	5 (7.8)	0.239
Cause of ESRD, n (%)			0.016			0.824
Glomerulonephritis	147 (55.7)	143 (42.8)		15 (31.9)	20 (31.3)	
Diabetes mellitus	34 (12.9)	58 (17.4)		10 (21.3)	15 (23.4)	
Hypertension	36 (13.6)	65 (19.5)		10 (21.3)	15 (23.4)	
Others	47 (17.8)	68 (20.4)		12 (25.5)	12 (18.8)	
Cold ischemic time (min)	255.4 ± 123.8	244.7 ± 127.3	0.321	257.8 ± 110.6	238.2± 113.6	0.383
HLA mismatch number	3.5 ± 1.6	3.7 ± 1.4	0.054	3.8 ± 1.7	3.9 ± 1.5	0.865
Induction, n (%)			<0.001			0.008
Basiliximab	216 (81.8)	221 (66.2)		38 (80.9)	36 (56.3)	
Anti-thymocyte globulin	48 (18.2)	113 (33.8)		9 (19.1)	28 (43.8)	
Main immunosuppressant						
Tacrolimus: Cyclosporine	247: 15	316: 18	0.327	44: 3	62: 2	0.649
PRA> 50%, n (%)	26 (19.4)	33 (15.5)	0.380	3 (10.7)	7 (16.7)	0.729

Values are expressed as means ± SDs, n (%).

Abbreviations: DDKT, deceased donor kidney transplantation; AKI, acute kidney injury; CVA, cerebrovascular accident; ESRD, end-stage renal disease, HLA, human leukocyte antigen; PRA, panel reactive antibody; CKD-EPI, chronic kidney disease-epidemiology collaboration; KDPI, kidney donor profile index

### Impact of AKI in DDs on the development of delayed graft function between young-DDKT and elderly-DDKT groups

**Figure 1:**
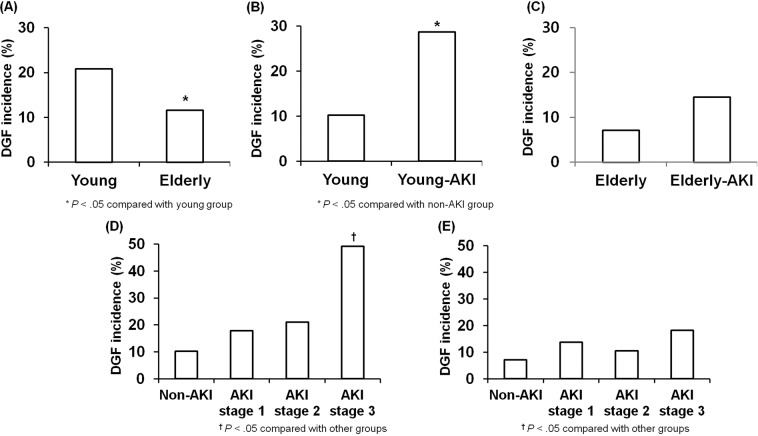
Comparison of the incidence of DGF (**A**) between young-DDKT and elderly-DDKT groups and between non-AKI-DDKT and AKI-DDKT subgroups within (**B**) young-DDKT and (**C**) elderly-DDKT groups. Comparison of the incidence of DGF according to the AKI stage in (**D**) young-DDKT and (**E**) elderly-DDKT groups. *p < 0.05 vs. young-DDKT, ^**†**^*p* < 0.05 vs. non-AKI.

The incidence of delayed graft function (DGF) was significantly higher in the young-DDKT group than that in the elderly-DDKT group (*p* < 0.05) (Fig. 1A). The incidence of DGF was significantly higher in the young-DDKT with AKI subgroup than that in the young-DDKT without AKI subgroup (*p* < 0.05) (Fig. 1B). However, there was no significant difference in the incidence of DGF between elderly-DDKT with AKI subgroup and elderly-DDKT without AKI subgroup (Fig. 1C). In the young-DDKT group, AKI stage 3 showed higher incidence of DGF than non-AKI, AKI stage 1, and AKI stage 2 (*p* < 0.05) (Fig. 1D). In the elderly-DDKT group, the incidence of DGF showed higher tendency in AKI stage 3 than non-AKI, AKI stage 1, and AKI stage 2, but it did not show statistical significance. (Fig. 1E). In the multivariable analysis, donor AKI was an independent risk factor for the development of DGF, adjusted by recipient age, recipient gender, donor gender, donor age, prior KT, induction and maintenance of immunosuppressant (hazard ratio: 3.604, 95% CI: 2.279–5.698, *p* < 0.001). However, donor age did not show any significance for the development of DGF (Table 2).

**Table 2:** Risk factors for development of delayed graft function in the deceased donor kidney transplantation.

Variables	Univariate analysis			Multivariable-adjusted analysis^a^		
	Exp (β)	95% C.I.	*p* value	Exp (β)	95% C.I.	*p* value
**Donor**						
Age at KT	0.998	0.985–1.010	0.708			
Female gender	0.611	0.397–0.942	0.026	0.776	0.472–1.275	0.317
Body mass index	1.011	0.957–1.068	0.403			
Hypertension	0.971	0.608–1.552	0.904			
Diabetes mellitus	0.591	0.275–1.274	0.180			
Cause of death - CVA	0.870	0.579–1.306	0.501			
Acute kidney injury	3.317	2.133–5.159	<0.001	3.604	2.279–5.698	<0.001
Cold ischemic time	1.001	1.000–1.003	0.056			
**Recipient**						
Age at KT	1.002	0.984–1.021	0.826			
Female gender	1.155	0.793–1.684	0.453			
Body mass index	0.982	0.933–1.034	0.490			
Hypertension	0.927	0.562–1.531	0.768			
Diabetes mellitus	1.136	0.722–1.790	0.581			
PRA (%)	1.005	0.998–1.011	0.147			
HLA mismatch number	1.020	0.899–1.158	0.758			
Tacrolimus (ref. CsA)	0.268	0.137–0.523	<0.001	0.141	0.065–0.303	<0.001
Basiliximab (ref. ATG)	1.293	0.863–1.935	0.213			

^a^Adjusted by recipient age, recipient gender, donor gender, donor age, prior KT, induction and maintenance immunosuppressant.

Abbreviations: DDKT, deceased donor kidney transplantation; CVA, cerebrovascular accident; PRA, panel reactive antibody; HLA, human leukocyte antigen; CsA, cyclosporine A; ATG, antithymocyte globulin.

### Impact of AKI in DDs on the change of allograft function between young-DDKT and elderly-DDKT groups

**Figure 2:**
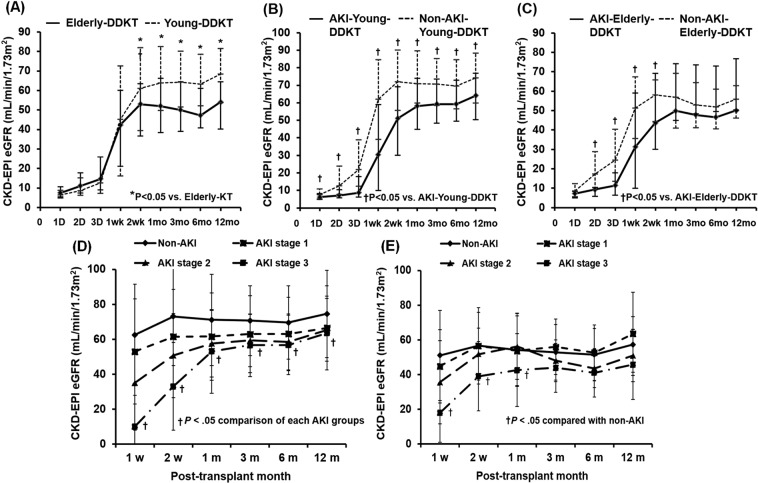
Comparison of the change in allograft function (eGFR by CKD-EPI) after KT (**A**) between young-DDKT and elderly-DDKT groups and between non-AKI-DDKT and AKI-DDKT subgroups within (**B**) young-DDKT and (**C**) elderly-DDKT groups. The change of allograft function according to AKI stage in (**D**) young-DDKT and (**E**) elderly-DDKT groups. *p < 0.05 vs. young-DDKT, ^†^*p* < 0.05 vs. non-AKI.

Allograft function between 2 weeks and 12 months post-KT was significantly lower in the elderly-DDKT group than that in the young-DDKT group (Fig. 2A). In the young-DDKT group, allograft function through the first 12 months post-KT was significantly lower in the AKI-young-DDKT subgroup than that in the non-AKI-young-DDKT subgroup (*p* < 0.05) (Fig. 2B). In the elderly-DDKT group, allograft function between 2 days and 2 weeks post-KT was significantly lower in the AKI-elderly-DDKT subgroup than that in the non-AKI-elderly-DDKT subgroup (*p* < 0.05), although it did not differ between the two subgroups at 1 year from KT (Fig. 2C). According to the AKI severity, those with AKI stage 3 had lower allograft function from 1 week after KT to the first 12 months post-KT in both young-DDKT group and elderly-DDKT group compared to those in the non-AKI subgroup (*p* < 0.05 respectively) (Figs. 2D,E). The incidence of biopsy-proven acute rejection (BPAR) within the first year after KT did not differ significantly between the young-DDKT group and the elderly-DDKT group or between two subgroups in young-DDKT or elderly-DDKT group.

### Impact of AKI in DDs on death-censored allograft survival between young-DDKT and elderly-DDKT groups

**Figure 3:**
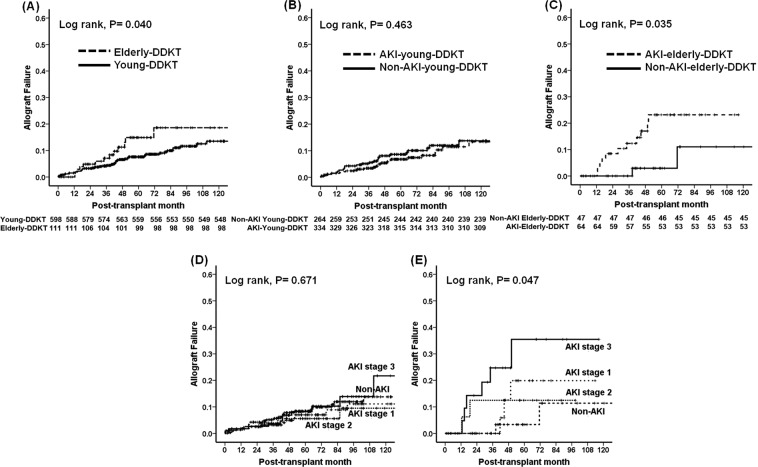
Comparison of death-censored allograft survival rate (**A**) between young and elderly-DDKT groups and between non-AKI-DDKT and AKI-DDKT subgroups within (**B**) young-DDKT and (**C**) elderly-DDKT groups. Death-censored allograft survival rate according to AKI stage in (**D**) young-DDKT and (**E**) elderly-DDKT groups.

A total of 71 (71/709, 10.0%) cases had graft failure, including 57 (8.0%) cases in the young-DDKT group (28 (3.9%) patients in the non-AKI-young-DDKT subgroup and 29 (4.1%) patients in the AKI-young-DDKT subgroup) and 14 (2.0%) in the elderly-DDKT group (3 (0.4%) patients in the non-AKI-elderly-DDKT subgroup and 11 (1.6%) patients in the AKI-elderly-DDKT subgroup). No significant difference was detected in the distribution of causes of allograft failure between the young-DDKT group and the elderly-DDKT group or between non-AKI-KT and AKI-KT subgroups within young-DDKT or elderly-DDKT group (Table 3). Death-censored allograft survival rate was significantly lower in the elderly-DDKT group than that in the young-DDKT group (*p* < 0.05) (Fig. 3A). In subgroup analysis, death-censored allograft survival rate was significantly lower in the AKI-elderly-DDKT subgroup than in the non-AKI-elderly-DDKT subgroup (*p* < 0.05) (Fig. 3C), although it was not significantly different between non-AKI-young-DDKT and the AKI-young-DDKT subgroups (Fig. 3B). There was no significant difference according to the AKI stage in the young-DDKT group (Fig. 3D), but AKI stage 3 showed the lowest death-censored allograft survival rate in the elderly-DDKT in comparison with non-AKI, stage 1, and stage 2 (*p* < 0.05) (Fig. 3E). In the multivariable analysis, AKI-elderly-DDKT was an independent risk factor for allograft failure (hazard ratio: 2.648, 95% CI: 1.170–5.994, *p* = 0.019) after adjusting by transplant years (1996~2005 vs. 2006~2010 vs. 2011~2017), transplant centers, recipient age, recipient gender, donor gender, DGF, and acute rejection. There was a significant interaction between AKI in DDs and elderly DDs for allograft failure (*p* for interaction = 0.002) (Table 4).

**Table 3:** Comparison of clinical outcomes according to acute kidney injury in young- or elderly donors.

Variables	Young DDKT			Elderly DDKT		
		AKI-KT	p for Trend	Non-AKI-KT	AKI-KT	p for Trend
Causes of graft failure, n (%)			0.275			1.000
Acute rejection	6 (21.4)	6 (20.7)		0	3 (27.2)	
Chronic allograft dysfunction	12 (42.9)	19 (65.5)		2 (66.7)	4 (36.4)	
Chronic antibody mediated rejection	1 (3.6)	0		0	1 (9.1)	
Recurrent glomerulonephritis	2 (7.1)	1 (3.4)		1 (33.3)	2 (18.2)	
BK virus-associated nephropathy	5 (17.9)	1 (3.4)		0	1 (9.1)	
Unknown	2 (7.1)	2 (7.0)		0	0	
Causes of death, n (%)			0.036			1.000
Cardiovascular disease	3 (15.8)	4 (22.2)		0	2 (33.3)	
Infection	5 (26.3)	8 (44.4)		2 (100.0)	3 (50.0)	
Malignancy	4 (21.1)	0		0	1 (16.7)	
Bleeding	2 (10.5)	1 (5.6)		0	0	
Hepatic failure	3 (15.8)	0		0	0	
Unknown	2 (10.5)	5 (27.8)		0	0	

Values are expressed as means ± SDs, n (%).

Abbreviations: DDKT, deceased donor kidney transplantation; AKI, acute kidney injury.

**Table 4:** Hazard ratios of allograft failure according to donor age (>60) or acute kidney injury.

	Unadjusted HR (95% C.I.)	P	Adjusted HR^a^ (95% C.I.)	P	P-value for interaction
Non-AKI-young DDKT	Reference		Reference		0.002
AKI-young DDKT	0.820 (0.488–1.380)	0.456	1.091 (0.615–1.935)	0.767	
Non-AKI-elderly DDKT	0.763 (0.231–2.517)	0.657	0.825 (0.190–3.583)	0.798	
AKI-elderly DDKT	2.462 (1.212–5.002)	0.013	2.648 (1.170–5.994)	0.019	

^a^Adjusted by transplant years (1996~2005 vs. 2006~2010 vs. 2011~2017), transplant centers, recipient age, recipient gender, donor gender, delayed graft function, acute rejection.

Abbreviations: AKI, acute kidney injury; DDKT, deceased donor kidney transplantation; HR, hazard ratio; C.I., confidence interval.

### Impact of AKI in DDs on patient survival between young-DDKT and elderly-DDKT groups

A total of 45 (45/709, 6.3%) patients died in this population. Among them, 37 (5.2%) patients were included in the young-DDKT group (19 (2.7%) patients in the non-AKI-young-DDKT subgroup and 18 (2.5%) patients in the AKI-young-DDKT subgroup) and 8 (1.1%) patients were included in the elderly-DDKT group (2 (0.3%) patients in the non-AKI-elderly-DDKT subgroup and 6 (0.8%) patients in the AKI-elderly-DDKT subgroup). There was no significant difference in the dispersion of the causes of patient death (Table 3). There was also no significant difference in patient survival rate between young-DDKT and elderly-DDKT groups, or between non-AKI-young-DDKT and AKI-young-DDKT subgroups, or between non-AKI-elderly-DDKT and AKI-elderly-DDKT subgroups (see Supplementary Fig. S2).

## Discussion

In this study, we investigated the impact of AKI occurred in elderly-DDs on the clinical outcomes after KT. As a result, we found that AKI itself did not show significant influence on long-term allograft outcomes (see Supplementary Fig. S3(A)). However, KT from elderly DDs with AKI showed worse long-term allograft survival than that from elderly DDs without AKI. This finding was not shown in transplant from young-DDs. Especially, KT from elderly DDs with AKI stage 3 showed worse long-term allograft survival than that other DDKT (see Supplementary Fig. S3(B)). Our results show that strategies to prevent or minimize the occurrence of AKI in DDs, especially in elderly, might be needed to improve long-term allograft outcomes.

First, we compared the clinical characteristics between young-DDs and elderly-DDs. Incidences of HTN and KDPI score were significantly higher in elderly-DDs. Since the presence of HTN and higher KDPI score could an suggest underlying chronic tissue injury, those donors could be diagnosed with CKD^17^. In the subgroup analysis, age and proportion of death due to CVA and BMI were significantly higher in the AKI-DD subgroup than in the non-AKI-DD subgroup of the young-DDKT group. It was difficult to see the direct-cause-result relationship because data were cross-sectional. However, higher BMI and CVA might be related to the development of AKI in these patients^18–20^.

Next, we compared short-term post-transplant clinical outcomes between young-DDKT and elderly-DDKT groups and between non-AKI and AKI subgroups. In consistent with our previous study^11,12,21^, the incidence of DGF was significantly higher or showed higher tendency when DDs had AKI irrespective of age of donor. In terms of the change of allograft function, allograft function at 1 year from KT showed lower value in the elderly-DDKT group than in the young-DDKT group. It may be because baseline capacity to recover is lower in high-KDPI subgroups due to pre-existed permanent functional loss of nephron in the elderly donor at baseline as we mentioned above^22–24^. In the subgroup analysis, the AKI-young-DDKT group showed lower allograft function at 1 year after KT than the non-AKI-young-DDKT group. As we mentioned above, it may be because AKI-young-DDKT showed older age and lower eGFR at baseline. Hence, their capacity to recover might be limited. In contrast, allograft function at 1 year after KT showed similar value between non-AKI-elderly-DDKT and AKI-elderly-DDKT subgroups. It might be because the baseline eGFR did not differ between the two subgroups. When we analyzed the change of allograft function until 1 year from KT according to AKI stage, although the allograft function was the lowest at 1 week after KT from DD with AKI stage 3 in both young-DDKT and elderly-DDKT groups, it showed similar values to those who took kidneys from DDs with stage 1 or 2 AKI at 1 year from KT. All these findings suggest that recently developed AKI in DDs might be a strong risk factor for DGF. For allograft function recovery at 1 year, baseline functional status could have a more significant impact^25^.

Our major issue is whether AKI in DDs has different influence on long-term allograft survivals between young-DDKT and elderly-DDKT groups. As we expected, AKI in DDs showed significant adverse effect on allograft survivals in elderly-DDKT, especially in AKI stage 3, but not in the young-DDKT group. Interestingly, the AKI-elderly-DDKT subgroup showed significantly worse allograft survival than the other three subgroups (non-AKI-elderly-DDKT, non-AKI-young-DDKT, and AKI-young-DDKT subgroups) (see Supplementary Fig. S1). In multivariable analysis using Cox regression hazard model, co-existence of AKI and elderly-DD was a significant risk factor for allograft failure. Both AKI in DDs and elderly DDs showed a significant interaction on allograft failure. However, either AKI in DDs or elderly in DD alone did not show significance in multivariable analysis as shown in previous reports including ours^11,12,14,26^. Finally, above findings could be explained by decreased functional nephron mass at baseline in kidney from elderly DDs^22–24^. In addition, development of inflammation by AKI beyond CKD state can accelerate the progression of kidney fibrosis. Hence, AKI in transplant naive patients can show more significant impact in elderly patients than in young patients in terms of a long term renal prognosis^15,16,27^.

In contrast to our expectation, the proportion of chronic allograft dysfunction as the cause of allograft failure was not different between non-AKI-elderly-DDKT and AKI-elderly-DDKT subgroups. At first, we expected that allograft failure due to progressive allograft injury after AKI might develop as non-specific “chronic allograft nephropathy” instead of immunologic process such as acute rejection or cAMR. However, although allograft rejection could be a direct cause of allograft failure in some patients, the probability for allograft failure can increase when it develops in kidney with decreased nephron mass such as kidney from elderly DDs. Another possible reason is that AKI can induce the activation of innate immune system. After transplant, it can be a crosstalk to the activation of allograft rejection^28–30^. Taken together, the main reason of the adverse allograft outcome in AKI-elderly-DDKT subgroup might be the low nephron mass at baseline. Hence, additional insult such as acute kidney injury before transplant and allo-immune responses after transplant may accelerate allograft failure.

In contrast to death-censored allograft survival rate, patient survival rate did not differ significantly between young-DDKT and elderly-DDKT groups or between AKI-DDKT and non-AKI-DDKT subgroups. It might be because patient death rate was so low that it cannot draw any conclusion about this issue. Another possible reason was that recipient age had such a strong effect on patient survival rate that the effect of other factors could not appear^31–34^. However, further evaluation should be done to clarify this interest.

Our study has some limitations as suggested in our previous reports using this cohort. First, because it was a retrospective study, our study had a possibility of selection bias. However, we analyzed a large number of KTRs from multiple centers. In addition, we also adjusted our results considering transplant centers and transplant year in the multivariable analysis. Second, we did not have detailed data or clinical outcomes of contralateral kidneys transferred to another transplant center according to the kidney allocation rule in Korea, which might have induced a bias during analysis. Well-designed prospective multi-center studies are required to overcome these issues. Lastly, we did not suggest any possible strategies to prevent or early detect AKI in elderly-DDs. In the recent, procurement biopsies are suggested as strategies to better detect grafts at high risk of worse outcomes for protecting against AKI in elderly-DDs to prevent its adverse effect on post-transplant allograft outcomes. Some research reported that procurement biopsies were performed in approximately one-half of all DD kidneys and 85% of expanded criteria donor/donor kidneys with high kidney donor profile index score in the United States, it was useful to predict the post-transplant allograft outcome^35^.

In conclusion, our study suggests that AKI occurred to elderly-DDs has a synergistically adverse effect on long-term allograft outcomes after KT in corresponding recipients. Therefore, careful monitoring and strategies against occurrence of AKI in DDs, especially in elderly-DDs, to prevent its adverse effect on post-transplant allograft outcomes.

## Materials and Methods

### Study population

**Figure 4:**
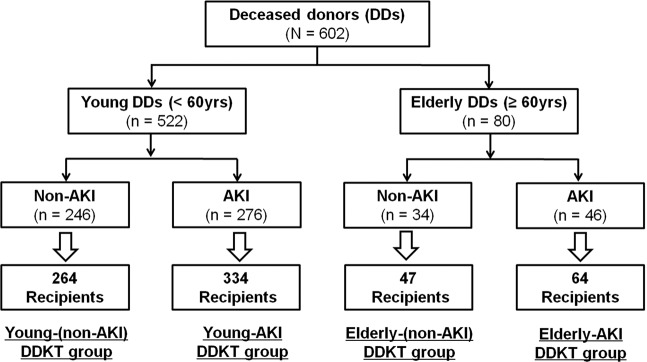
Patient algorithm and distribution in this study. DDs were classified into elderly DDs and young DDs based on an age of 60. Each group was divided into AKI-DD and non-AKI-DD subgroup according to the diagnosis of AKI by KDIGO criteria. Finally, KTRs belonged to one of following four subgroups: non-AKI-young-DDKT, AKI-young-DDKT, non-AKI-elderly-DDKT, and AKI-elderly-DDKT subgroups according to their corresponding donors.

A total of 709 KTRs receiving kidneys from 602 DDs at four transplant centers between October 1996 and December 2017 were enrolled. Young and elderly DDs were defined according to the age at the time of donation (<60 years vs. ≥ 60 years). AKI in DDs was defined according to the Kidney Disease: Improving Global Outcomes (KDIGO) criteria as described in previous reports^12,36^. Patient distribution is presented in Fig. 4.

KTRs were classified into young-DDKT and elderly-DDKT groups. Each group was subdivided into non-AKI-DDKT and AKI-DDKT subgroups according to whether their corresponding DDs were young or elderly and whether they were diagnosed to AKI or not. Out of 602 DDs, 522 DDs were young DDs and the remaining 80 were Elderly-DDs. In the group of young DDs, AKI was diagnosed in 276 (52.9%). In the group of elderly DDs, 46 (57.5%) donors had AKI. Finally, 598 cases of DDKT were classified into young-DDKT group and 111 cases were classified into elderly-DDKT group. Within the young-DDKT group, 264 cases were classified as the non-AKI-Young-DDKT subgroup and 334 cases were classified in the AKI-Young-DDKT subgroup. Within the elderly-DDKT group, 47 cases were classified as the non-AKI-Elderly-DDKT subgroup, and 64 cases were classified as the AKI-Elderly- DDKT subgroup.

### Clinical parameters and outcomes

We analyzed the medical records of both DDs and their corresponding DDKT recipients in the study population retrospectively. We collected baseline donor data including age, sex, body mass index (BMI) (kg/m^2^), history of diabetes mellitus (DM) and hypertension (HTN), cause of death due to cerebrovascular accident (CVA), and kidney donor profile index score (KDPI)^37–39^. Data of baseline and followed-up estimated glomerular filtration rate (eGFR) were calculated using the Chronic Kidney Disease Epidemiology Collaboration (CKD-EPI) equation to assess kidney function of DDs daily between the admission date and the date of KT. Baseline eGFR was the value obtained when DDs were admitted to the hospital for another cause and determined to brain death.

In addition, we collected the baseline recipient data: age, sex, BMI, dialysis type, dialysis vintage before KT, frequency of previous KTs, cause of end-stage renal disease, history of DM and HTN, cold ischemic time, number of human leukocyte antigen (HLA) mismatches, immunosuppressant type for induction, and maintenance and percentage of panel-reactive antibodies (PRAs).

BPAR was diagnosed by Banff classification^40^. DGF was defined as the need for dialysis within the first week after KT because of unrecovered allograft function^41^. Death-censored allograft survival rate was defined as the rate from KT to the return to dialysis excepting for allograft loss by patient death. Patient survival rate was defined as the rate from KT to patient death by any cause.

The primary outcome of this study was to investigate death-censored allograft survival rate of KTRs by young-DDs or elderly-DDs with AKI. Death-censored allograft survival was compared between the non-AKI-DDKT and AKI-DDKT groups with KT from young DDs or elderly DDs, respectively. The interaction between donor age and AKI was also analyzed. Secondary outcomes were to investigate the incidence of DGF, BPAR, the change of allograft function during the first year after KT, patient survival rate between young-DDKT and elderly-DDKT, and patient survival rate between non-AKI-DDKT and AKI-DDKT subgroups in young-DDKT and elderly-DDKT groups. Clinical outcomes were also analyzed according to AKI severity in each group analysis. The cause of allograft failure included biopsy-proven acute rejection (both T-cell mediated rejection and antibody-mediated rejection (AMR)), biopsy-proven chronic AMR (cAMR), chronic allograft dysfunction, biopsy-proven BK virus-associated nephropathy (BKVN), and biopsy-proven recurrent primary glomerulonephritis. Chronic allograft dysfunction was defined when allograft findings showed non-specific chronic tissue injury without evidence of rejection or when allograft biopsy was not done within one year of allograft failure and the allograft function showed a gradual deterioration several years before the allograft failure.

This study was approved by Institutional Review Boards of Seoul St. Mary’s Hospital (XC15RIMI0061K), Uijeongbu St. Mary’s Hospital (XC15RIMI0061U), Keimyung University School of Medicine, Dongsan Medical Center (2019-09-053), and Daejeon St. Mary’s Hospital (XC15RIMI0061K). The requirements for informed consent were waived by the Institutional Review Boards of above four centers because it was explained to all donor’s family and all recipients prior to KT that personal data associated with the donor and recipient’s clinical course was used and the information identifying the individual were protected. Our study did not contain any distinguishable personal identification information as a retrospective medical record study. Furthermore, all methods were performed in accordance with the relevant guidelines and regulations. The above four transplant centers never underwent transplantation with kidneys procured from prisoners, and this study did not include them as a study population.

### Statistical analysis

Continuous variables with normal distributions were expressed as mean with standard deviation and analyzed by Student’s t-test. While those with non-normal distributions were expressed as median with interquartile range and analyzed by Mann-Whitney test. Categorical variables were expressed as number with percentage and analyzed by Chi-square test or Fisher’s exact test. Death-censored graft survival and patient survival rates were investigated by Kaplan-Meier curves and log-rank tests. All missing data were treated by censoring since the last follow-up date. Cox proportional hazards regression model was used to investigate an independent risk factor for allograft failure. Transplant years (1996~2005 vs. 2006~2010 vs. 2011~2017), transplant centers, recipient age, recipient gender, donor gender, DGF, and BPAR were contained as confounding factors. The effect of interactions between AKI on DDs and elderly DDs were explored by adding interaction terms to the model where donor age groups were treated as ordinal numbers across the entire population. When the p value was less than 0.05, it was considered statistically significant. Statistical tool was used by SPSS 19.0 software (SPSS Inc., Chicago, IL, USA).

## Supplementary information

Supplementary Information.

## Data Availability

All data generated or analyzed during this study are included in this published article and its Supplementary Information files. The datasets generated during and/or analyzed during the current study are available from the corresponding author on reasonable request.
