# Supplementary material for: Impact of acute kidney injury in elderly versus young deceased donors on post-transplant outcomes: A multicenter cohort study
Source: Sci Rep. 2020 Feb 28;10:3727. doi: 10.1038/s41598-020-60726-8 (PMC7048728; doi:10.1038/s41598-020-60726-8)

**Impact of acute kidney injury in elderly versus young deceased donors on post-transplant outcomes: A multicenter cohort study**

Woo Yeong Park^8,9^, (ORCID; 0000-0003-2662-2898), Jeong Ho Kim^3^, Eun Jung Ko^1,2^, Ji-Won Min^1,4^, Tae Hyun Ban^1,5^, Hye-Eun Yoon^1,6^, Young Soo Kim^1,7^, Kyubok Jin^8,9^, Seungyeup Han^8,9^, Chul Woo Yang^1,2^ and Byung Ha Chung^1,2^*****

*^1^Transplant research center, ^2^Division of Nephrology, Department of Internal Medicine, Seoul St. Mary's Hospital, College of Medicine, The Catholic University of Korea, Seoul, Republic of Korea. ^3^Division of Nephrology, Department of Internal Medicine, Daejeon St. Mary’s hospital, College of Medicine, The Catholic University of Korea, Daejeon, Republic of Korea. ^4^Division of Nephrology, Department of Internal Medicine, Bucheon St. Mary’s hospital, College of Medicine, The Catholic University of Korea, Bucheon, Republic of Korea. ^5^Division of Nephrology, Department of Internal Medicine, Eunpyeong St. Mary’s hospital, College of Medicine, The Catholic University of Korea, Seoul, Republic of Korea. ^6^Division of Nephrology, Department of Internal Medicine, Incheon St. Mary’s hospital, College of Medicine, The Catholic University of Korea, Incheon, Republic of Korea. ^7^Division of Nephrology, Department of Internal Medicine, Uijeongbu St. Mary’s hospital, College of Medicine, The Catholic University of Korea, Uijeongbu, Republic of Korea. ^8^Department of Internal Medicine, Keimyung University School of Medicine, Daegu, Republic of Korea. ^9^Keimyung University Kidney Institute, Daegu, Republic of Korea.*

**Corresponding author; Byung Ha Chung, MD**

Department of Internal Medicine, Seoul St. Mary’s Hospital, 505 Banpo-Dong, Seocho-Ku, 137-040, Seoul, Korea. Fax: +82-2-536-0323, Phone: +82-2-2258-6066, **E-mail;** chungbh@catholic.ac.kr

**Supplementary Table S1. Comparison of clinical and laboratory parameters between young-DDKT group and elderly-DDKT group**

| **Variable** | **Young-DDKTs** | **Elderly-DDKT** | ***P-*value** |
| --- | --- | --- | --- |
| **Donors** | n=522 | n=80 |  |
| Age at KT (years) | 41.9 ± 13.3 | 63.9 ± 3.6 | <0.001 |
| Gender (Male:Female) | 360:162 | 53:27 | 0.608 |
| Body mass index (kg/m^2^) | 23.2 ± 3.7 | 23.4 ± 2.9 | 0.657 |
| Hypertension, n (%) | 88 (17.0) | 33 (41.8) | < 0.001 |
| Diabetes mellitus, n (%) | 42 (8.1) | 12 (15.2) | 0.056 |
| Cause of donor death – CVA, n (%) | 366 (70.1) | 58 (72.5) | 0.240 |
| Baseline eGFR (ml/min/1.73m^2^)  (CKD-EPI) | 84.1 ± 25.1 | 75.9 ± 16.4 | 0.001 |
| GFR at allocation (ml/min/1.73m^2^)  (CKD-EPI) | 67.9 ± 41.4 | 52.2 ± 31.6 | < 0.001 |
| KDPI score (%) | 58.6 ± 22.4 | 93.2 ± 5.3 | < 0.001 |
| AKI, n (%) |  |  | 0.712 |
| Stage 1 | 124 (23.8) | 18 (22.5) |  |
| Stage 2 | 69 (13.2) | 12 (15.0) |  |
| Stage 3 | 83 (15.9) | 16 (20.0) |  |
| **Recipient** | n = 598 | n = 111 |  |
| Transplant year, n (%) |  |  | 0.031 |
| 1996 ~ 2005 | 36 (6.0) | 5 (4.5) |  |
| 2006 ~ 2010 | 95 (15.9) | 7 (6.3) |  |
| 2011 ~ 2017 | 467 (78.1) | 99 (89.2) |  |
| Age at KT (year) | 46.7 ± 8.5 | 63.6 ± 3.0 | <0.001 |
| Gender (Male:Female) | 349:249 | 69:42 | 0.465 |
| Body mass index (kg/m^2^) | 23.1 ± 3.9 | 23.4 ± 3.1 | 0.528 |
| Hypertension, n (%) | 500 (83.6) | 96 (86.5) | 0.572 |
| Diabetes mellitus, n (%) | 113 (18.9) | 30 (27.0) | 0.054 |
| Dialysis duration, years | 8.6 ± 11.1 | 6.9 ± 4.1 | 0.114 |
| Previous KT, n (%) | 71 (11.9) | 6 (5.4) | 0.046 |
| Cause of ESRD, n (%) |  |  | 0.127 |
| Glomerulonephritis | 290 (48.5) | 35 (31.5) |  |
| Diabetes mellitus | 92 (15.4) | 27 (24.3) |  |
| Hypertension | 101 (16.9) | 25 (22.5) |  |
| Others | 115 (19.2) | 24 (21.6) |  |
| Cold ischemic time (min) | 250.8 ± 123.5 | 251.8 ± 127.9 | 0.941 |
| HLA mismatch number | 3.6 ± 1.5 | 3.7 ± 1.6 | 0.568 |
| Induction, n (%) |  |  | 0.169 |
| Basiliximab | 437 (73.1) | 74 (66.7) |  |
| Anti-thymocyte globulin | 161 (26.9) | 37 (33.3) |  |
| Major immunosuppressant  Tacrolimus : Cyclosporine | 563 : 33 | 106 : 5 | 0.872 |
| PRA > 50 %, n (%) | 59 (17.0) | 10 (14.3) | 0.724 |

Values are expressed as means ± SDs, n (%). eGFR is calculated using CKD-EPI formula.

DDKT, deceased donor kidney transplantation; CVA; cerebrovascular accident; eGFR, estimated glomerular filtration rate; CKD-EPI, Chronic Kidney Disease Epidemiology Collaboration; AKI, acute kidney injury; ESRD, end-stage renal disease; HLA, human leukocyte antigen; PRA, panel reactive antibody; KDPI, kidney donor profile index

**Supplementary Figure S1.** Comparison of death-censored graft survival rate among non-AKI-young-DDKT, AKI-young-DDKT, non-AKI-elderly-DDKT, and AKI-elderly-DDKT subgroups.


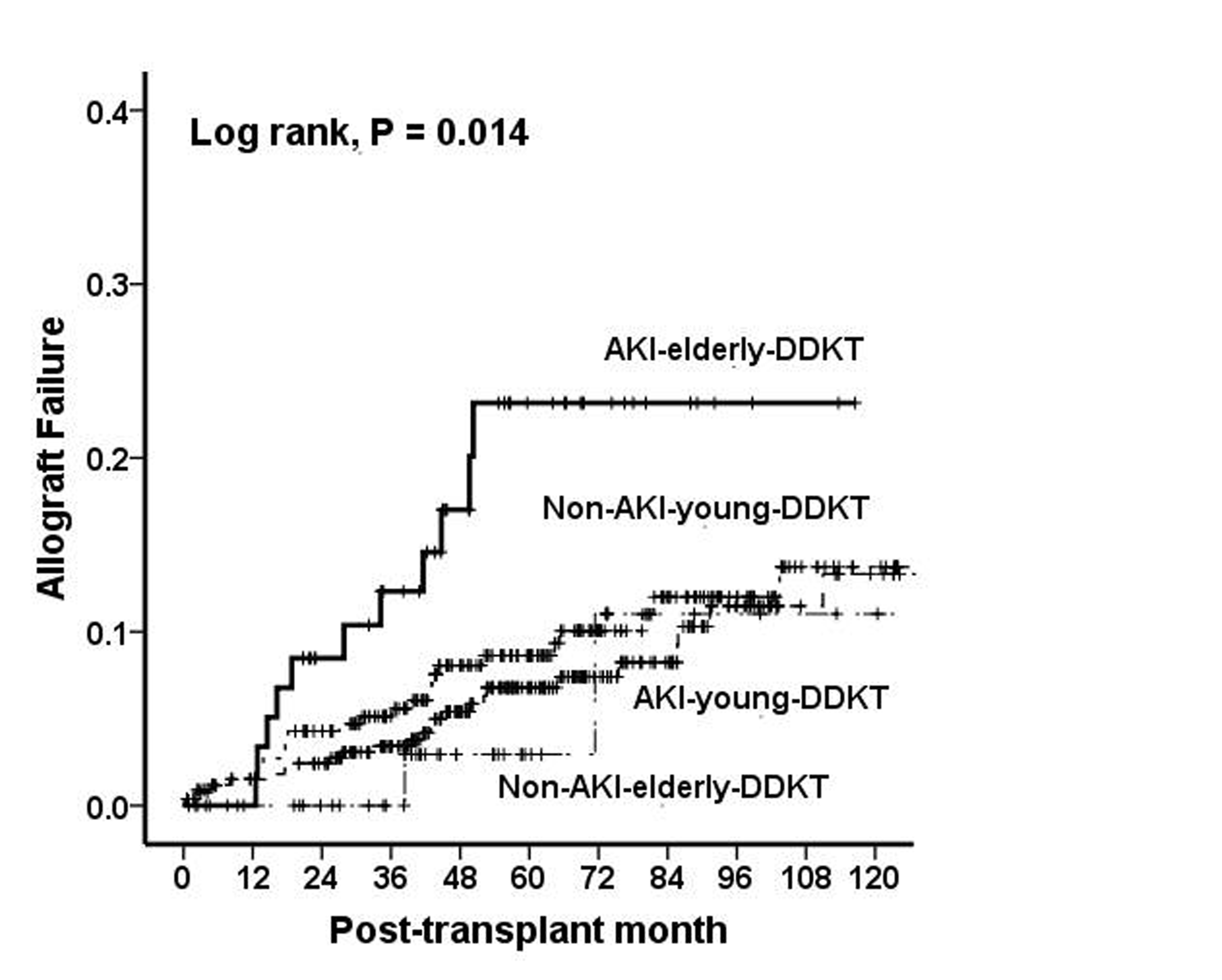


**Supplementary Figure S2.** Comparison of patient survival rate **(A)** between young-DDKT and elderly-DDKT groups and between non-AKI-DDKT and AKI-DDKT subgroups within **(B)** young-DDKT and **(C)** elderly-DDKT groups.


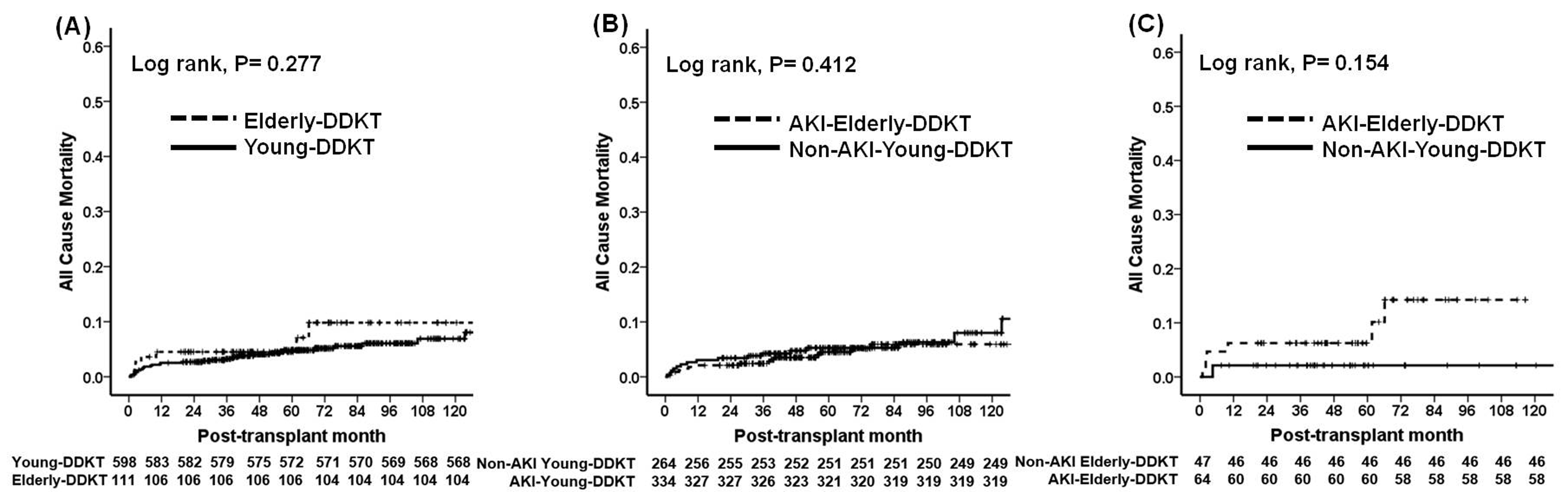


**Supplementary Figure S3.** Comparison of death-censored graft survival rate (A) between non-AKI-DDKT and AKI-DDKT and (B) between other DDKT and AKI stage 3-elderly-DDKT groups.


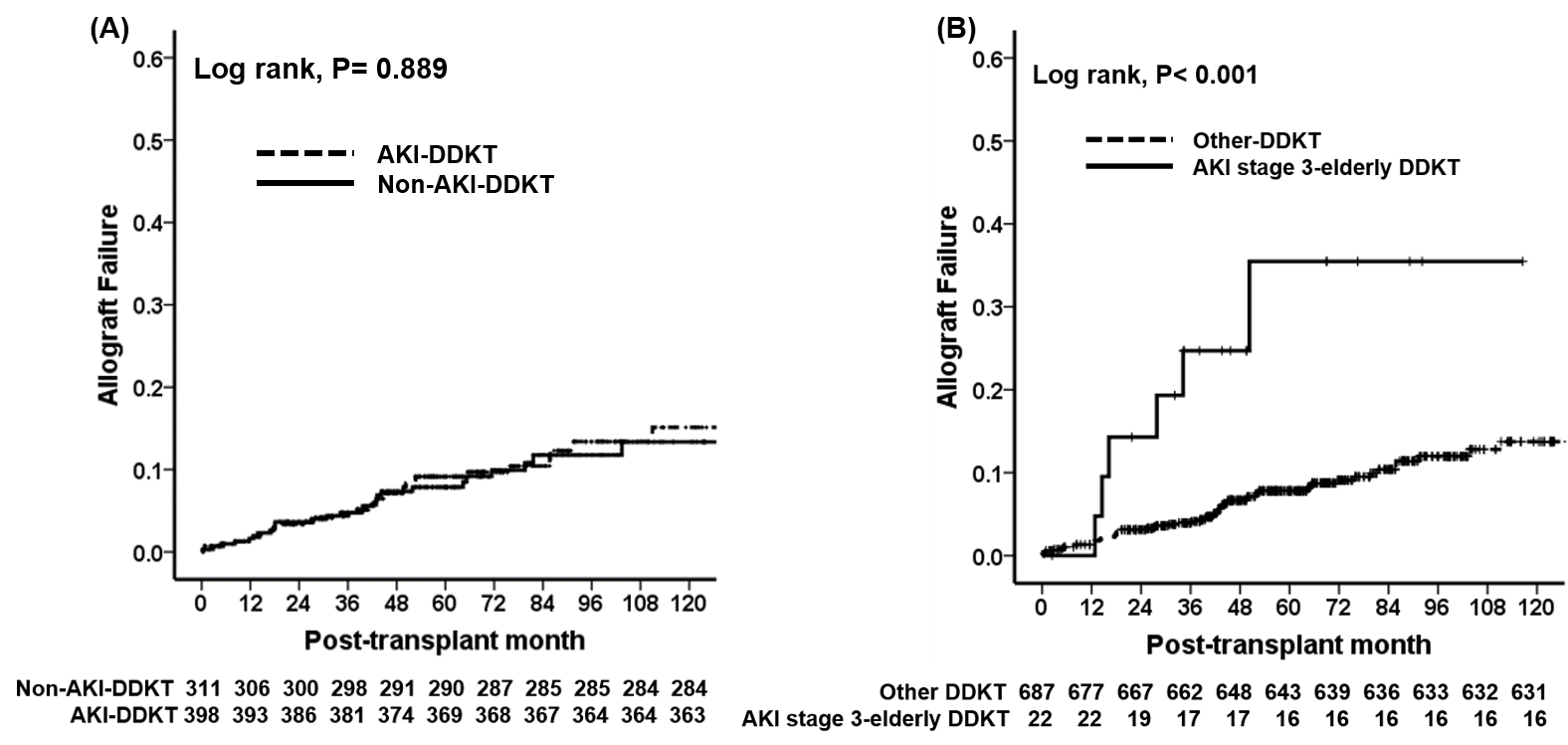

Supplement: Supplementary file 1 — Supplementary Information. [file 41598_2020_60726_MOESM1_ESM.doc]
